# Reaching Populations at Risk for HIV Through Targeted Facebook Advertisements: Cost-Consequence Analysis

**DOI:** 10.2196/38630

**Published:** 2023-01-20

**Authors:** John J Hanna, Sameh N Saleh, Christoph U Lehmann, Ank E Nijhawan, Richard J Medford

**Affiliations:** 1 Clinical Informatics Center University of Texas Southwestern Medical Center Dallas, TX United States; 2 Division of Infectious Diseases and Geographic Medicine University of Texas Southwestern Medical Center Dallas, TX United States; 3 Department of Bioinformatics University of Texas Southwestern Medical Center Dallas, TX United States; 4 Department of Population and Data Sciences University of Texas Southwestern Dallas, TX United States; 5 Department of Pediatrics University of Texas Southwestern Dallas, TX United States

**Keywords:** human immunodeficiency virus, social media, Facebook, health behavior, health care seeking behavior, consumer health, HIV diagnosis, HIV testing

## Abstract

**Background:**

An undiagnosed HIV infection remains a public health challenge. In the digital era, social media and digital health communication have been widely used to accelerate research, improve consumer health, and facilitate public health interventions including HIV prevention.

**Objective:**

We aimed to evaluate and compare the projected cost and efficacy of different simulated Facebook (FB) advertisement (ad) approaches targeting at-risk populations for HIV based on new HIV diagnosis rates by age group and geographic region in the United States.

**Methods:**

We used the FB ad platform to simulate (without actually launching) an automatically placed video ad for a 10-day duration targeting at-risk populations for HIV. We compared the estimated total ad audience, daily reach, daily clicks, and cost. We tested ads for the age group of 13 to 24 years (in which undiagnosed HIV is most prevalent), other age groups, US geographic regions and states, and different campaign budgets. We then estimated the ad cost per new HIV diagnosis based on HIV positivity rates and the average health care industry conversion rate.

**Results:**

On April 20, 2021, the potential reach of targeted ads to at-risk populations for HIV in the United States was approximately 16 million for all age groups and 3.3 million for age group 13 to 24 years, with the highest potential reach in California, Texas, Florida, and New York. When using different FB ad budgets, the daily reach and daily clicks per US dollar followed a cumulative distribution curve of an exponential function. Using multiple US $10 ten-day ads, the cost per every new HIV diagnosis ranged from US $13.09 to US $37.82, with an average cost of US $19.45. In contrast, a 1-time national ad had a cost of US $72.76 to US $452.25 per new HIV diagnosis (mean US $166.79). The estimated cost per new HIV diagnosis ranged from US $13.96 to US $55.10 for all age groups (highest potential reach and lowest cost in the age groups 20-29 and 30-39 years) and from US $12.55 to US $24.67 for all US regions (with the highest potential reach of 6.2 million and the lowest cost per new HIV diagnosis at US $12.55 in the US South).

**Conclusions:**

Targeted personalized FB ads are a potential means to encourage at-risk populations for HIV to be tested, especially those aged 20 to 39 years in the US South, where the disease burden and potential reach on FB are high and the ad cost per new HIV diagnosis is low. Considering the cost efficiency of ads, the combined cost of multiple low-cost ads may be more economical than a single high-cost ad, suggesting that local FB ads could be more cost-effective than a single large-budget national FB ad.

## Introduction

### Undiagnosed HIV

Complications and risks from undiagnosed HIV infections continue to be a public health challenge [1]. In 2020, a total of 16% of people with HIV worldwide were unaware of their HIV status, and 27% were not receiving antiretroviral therapy [2]. In the United States, undiagnosed HIV has an estimated prevalence of 13.3%, with certain populations affected disproportionately [3]. The latest estimates from the Centers for Disease Control and Prevention (CDC) indicate that effective HIV prevention and treatment are not adequately reaching men who have sex with men, transgender persons, American Indian, Alaska Native, Native Hawaiian, other Pacific Islander, African Americans, Asians, Hispanics, and youth [4,5]. In 2019, an estimated 44.3% of people aged between 13 to 24 years were unaware of their HIV infection [5]. Moreover, during the COVID-19 pandemic and the resulting shutdowns, fewer HIV tests were performed [6], triggering concerns that unprecedented financial stressors to patients and health care systems and required modifications to health care delivery greatly disrupted HIV diagnosis and care [7].

### Social Media as a Health Communication Solution

In the last decade, social media has provided health communication solutions for multiple health challenges, including patient outreach and education. Despite concerns about privacy and data use [8], social media platforms and medical crowdfunding websites have proven to be powerful tools for health communication in the social media era [9-13]. Compared with the beginning of the HIV epidemic, the COVID-19 pandemic highlighted social media solutions and challenges such as the rapid spread of misinformation [9-15]. Dis- and misinformation require social media companies to step up their stewardship by removing false information and redirecting users to reputable websites. In this study, we evaluated the potential benefits of social media by estimating the projected cost and efficacy of using Facebook (FB) advertisements (ads)—the biggest social network outreach solution—to reach populations at risk for HIV.

Meta Inc (Menlo Park) continues to lead the social media market with 2.91 billion active users monthly as of the fourth quarter of 2021 [16]. The FB ad platform allows promoters to target audiences that meet certain criteria, including specific gender, age, demographics, interests, and location [17]. Previous studies have tailored campaigns to target populations of interest, including underrepresented populations. Although some studies have evaluated the efficacy and cost-effectiveness of the FB platform [18-23], others have compared the cost-effectiveness of FB ads with other ads [24,25].

### Previous Use of FB Ads to Reach Populations at Risk for HIV

The Chicago pre-exposure prophylaxis (PrEP) campaign (PrEP4L) was launched in 2016 and used the FB ad platform to disseminate nonstigmatizing health education to high-risk populations. PrEP4L garnered 6,970,127 views on FB and 1,719,446 views on Instagram. The average number of visitors to the PrEP4L website from this campaign was 182 per day, with a click through rate (CTR) of 0.06%, which is below the industry standard of 0.5% to 0.9% for social campaigns [22]. Another social media study recruited individuals identified as lesbian, gay, bisexual, and transgender (LGBT) through ads on FB and Instagram in 2016 and found that study enrollment was notably faster on social media than in-person recruitment in LGBT bars and night clubs [20]. However, gay women, bisexual men and women, and other gender minorities were easier to recruit than gay men via social media [20]. These studies highlight the feasibility of reaching LGBT populations on FB with an opportunity for improvement in terms of cost-effectiveness and reaching gay men.

### Use of FB Ad Estimates During the Ad Creation Process

Researchers used FB-provided estimates during ad creation to demonstrate that FB ad audience estimates can be used to model regional variations in the prevalence of health conditions such as obesity [26-28]. During the process of FB ad creation and based on the selected target criteria, FB ads provide promoters with a projected potential reach that estimates the size of the audience matching the selected target criteria. The potential reach depends on the target criteria and the ad placement options selected while creating an ad [29]. On the basis of the user-adjusted budget, the platform estimates the daily reach and daily clicks.

### Study Aim

In this US-centric study, we evaluated and compared the projected cost and efficacy of different FB ad approaches, simulating a 10-day video ad campaign that targets at-risk populations for HIV to estimate the costs for every resulting new HIV diagnosis using the health care industry conversion rate [30] and HIV positivity rates [31] in various regions and age groups.

## Methods

### Study Definitions

In this study, we refer to FB ad metrics as defined by FB [29] (definitions summarized in Table 1). We refer to the US regions as defined by the US Census Bureau and as referenced in the CDC report “Diagnosis of HIV Infection in the US and Dependent Areas, 2019” [3]. We created ads (without actually launching them) targeting only Puerto Rico and the US Virgin Islands and referred to these territories as US dependent areas. For our estimations, we used simulated FB ad estimates, HIV positivity rates provided by the CDC [31], and the average conversion rate for the health care industry on FB (11%) as reported by previous marketing research [30].

**Table 1:** Study definitions.

Term	Definition
Detailed targeting	An option available during FB^a^ ad^b^ creation that allows promoters to define the group of people that will see an ad on FB. It may include other pages or ads users click on and activities they engage with on FB. Additional selection criteria include demographics such as age, gender, location, and network connection speed.
Ad goal	A choice that promoters make when creating an ad to share with FB to influence the result they receive from the promotion. Promoters can also select an “automatic” goal by allowing FB to set the most relevant goal based on other ad settings.
Estimated potential reach	The estimated maximum audience size that could see an ad based on the selected target criteria, ad placements, and how many people were shown ads on FB apps and services in the past 30 d. Potential reach is not an estimate of how many people will actually see the ad and may change with time. Estimates are not designed to match census population.
Estimated daily reach	The estimated number of people an FB ad will reach in a certain audience each day based on budget and ad bid. Ad bid is how much an advertiser is willing to pay for a specific action.
Estimated daily link clicks	The estimated number of link clicks that an FB ad will receive each day based on a campaign performance and estimated daily reach.
Cost per 1000 people reached	The average cost to reach 1000 people with an FB ad; reach can be a more insightful metric than impressions, because it measures how many people were exposed to an ad and how efficiently an ad reached them.
Conversion rate	The percentage of users who perform a desired action after clicking on an ad.

^a^FB: Facebook.

^b^ad: advertisement.

### Ad Creation Process for Age Group 13 to 24 Years

**Figure 1:**
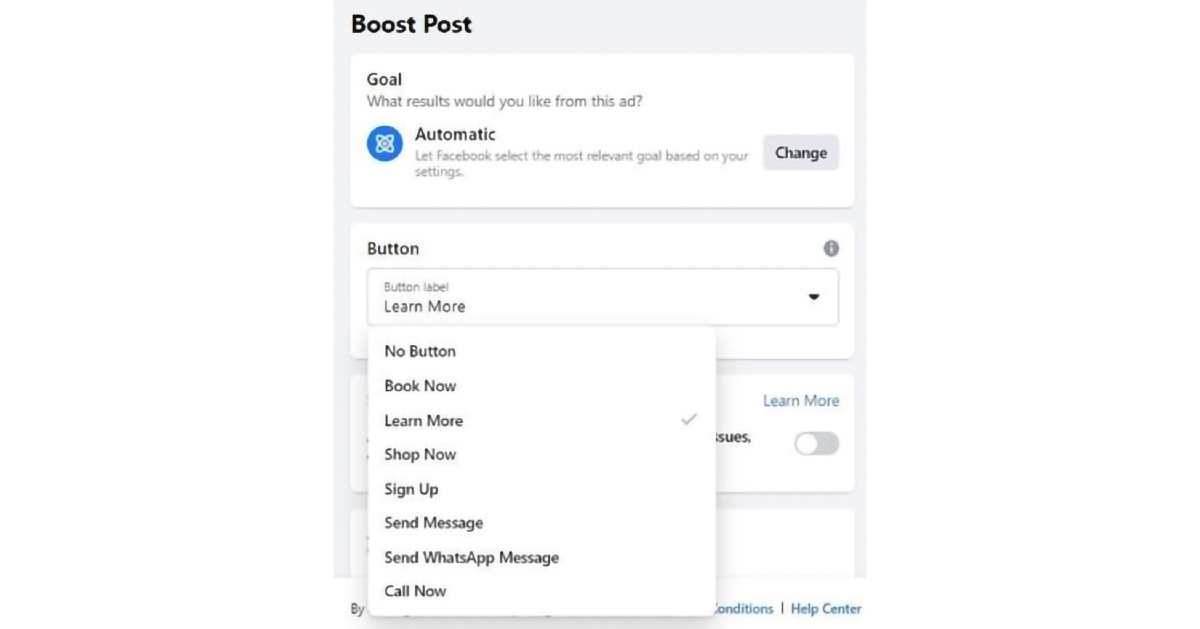
“Facebook Ads platform screenshot”—an example of post boosting with automatic goal that lets Facebook target the most relevant advertiser goal and the button label selection “learn more”.

**Figure 2:**
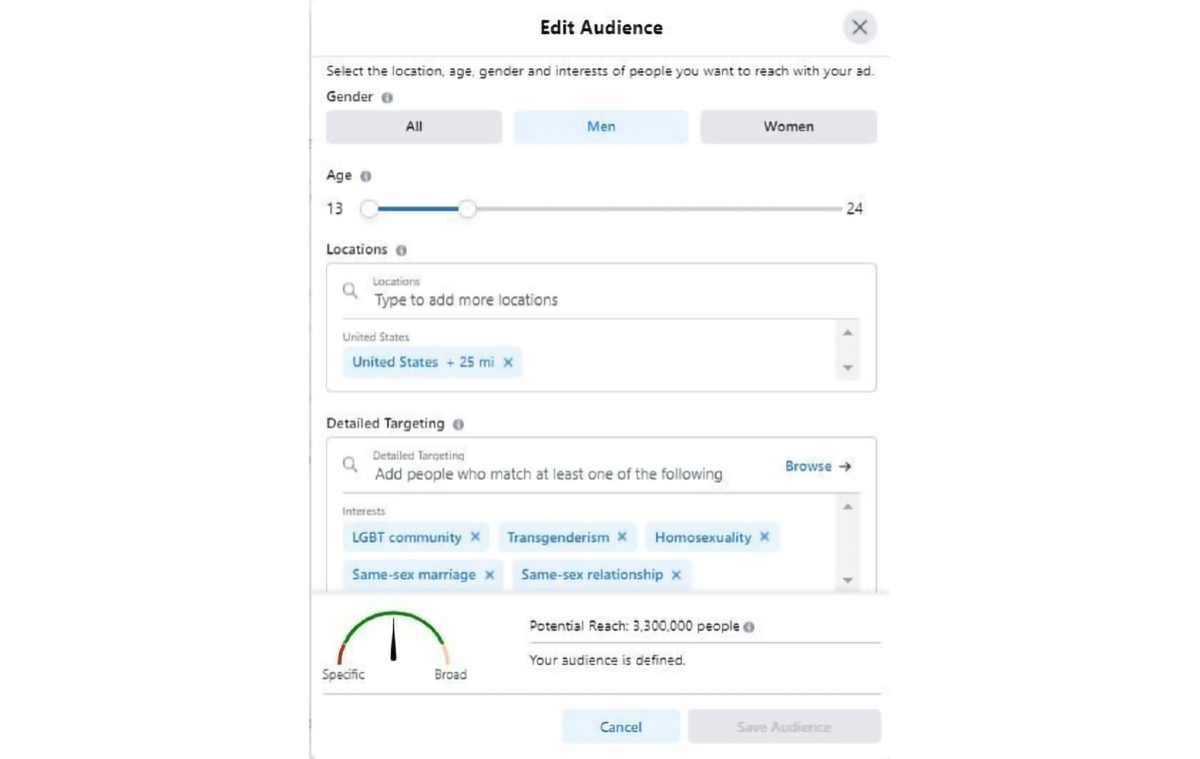
“Facebook Ads platform screenshot”—The Facebook Ads platform allows users to select gender, age, location, and other detailed target criteria based on demographics, interests, behaviors, and more. It also provides potential reach of the defined target audience.

We created an FB video ad for a 10-day period without launching it. First, we selected the FB ad goal (Figure 1). The ad’s target criteria included participants being men, aged 13 to 24 years (the age group with the highest undiagnosed HIV rates), and with 1 or more of the following interests as defined by FB: “homosexuality, same-sex marriage, same-sex relationship, transgenderism, or LGBT community” (Figure 2). On changing the target geographic location, FB ads provide an estimated potential reach in that location. As estimates vary over time, we collected the potential reach for each state and for the United States as a whole for the same single ad on the same day (April 20, 2021).

**Figure 3:**
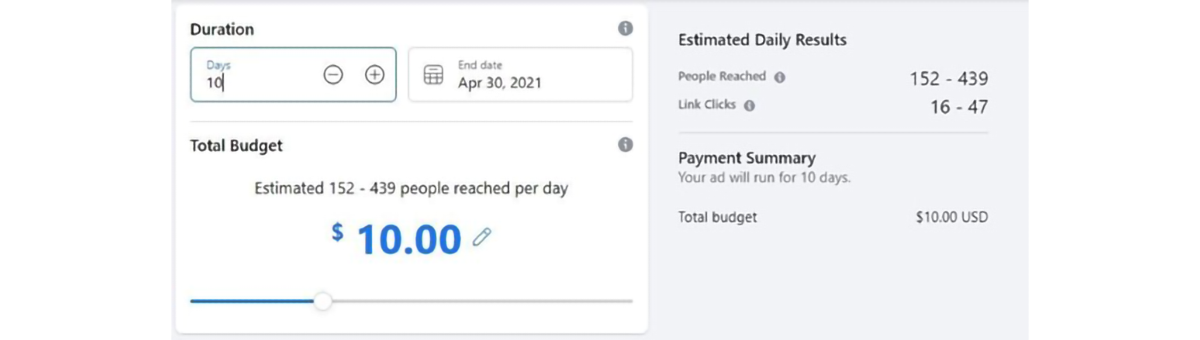
“Facebook Ads platform screenshot”—an example of adjusting the Facebook Ads campaign settings to 10 days using US $10 total budget. Facebook provides estimated people reached per day and estimated daily link clicks.

FB ads allow promoters to choose a budget, and based on the budget, FB provides an estimated daily reach and estimated daily number of link clicks. For each state, we adjusted the budget to reach the total estimated target population in 10 days, which meant that we targeted an estimated 10% of the total potential reach per day. We adjusted our budget to the nearest US $10 to achieve this goal. We documented the budget, estimated daily reach, and estimated daily clicks for 2 manually adjusted budgets for each ad campaign. The first budget was adjusted to target the highest estimated reach over 10 days, and the second budget was adjusted to target the lowest estimated reach over 10 days (Figure 3).

### Ad Estimates at Different Budgets

We collected the estimated daily reach and daily clicks for a single ad with the same criteria targeting the entire United States at multiple budget set points ranging from US $10 to US $1,000,000 to explore the differences in reach and link clicks at different budget levels.

### Estimating Ad Cost per New HIV Diagnosis

**Figure 4:**
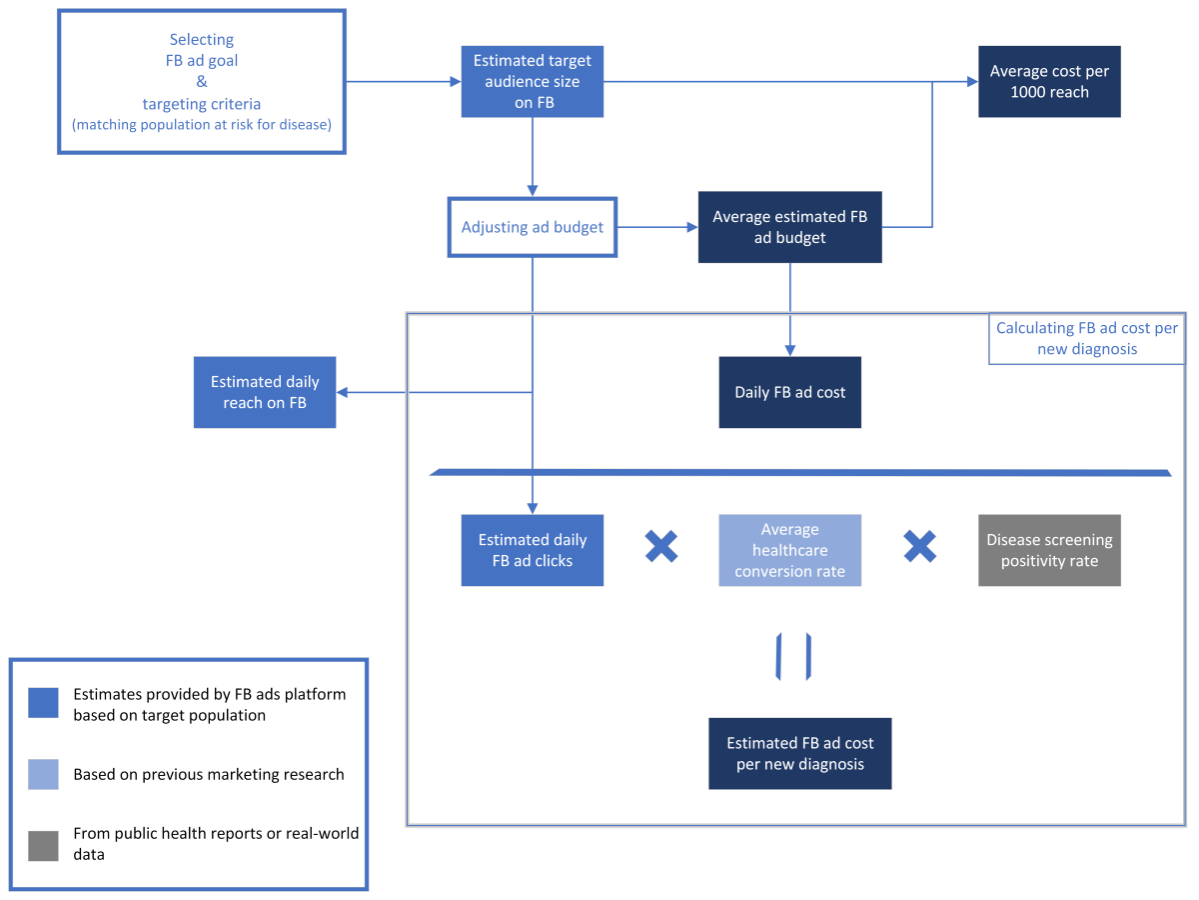
Proposed framework to evaluate cost and efficacy of Facebook (FB) advertisements (ads) to reach population at risk for diseases with health promotion ads for disease screening.

Finally, for every state, we estimated the cost of ads for every new HIV diagnosis secondary to the campaign based on estimated daily clicks (provided by FB), the health care industry conversion rate [27], and previously reported new HIV diagnosis positivity rates [28] (Figure 4). We also compared the ad’s cost for every new diagnosis using a single ad in the United States as a whole the ad’s cost using multiple US $10 ads in different US regions or states and age groups (Multimedia Appendix 1 [32]).

### Ethical Considerations

As all the data used in our study were publicly available, no institutional review board approval was required. All FB ad estimates used for modeling the cost per new HIV diagnosis were collected from the provided anonymous estimates on the FB ad platform without launching any ads. Hence, as no simulated study ads reached consumers, informed consent was not required. FB was not a collaborator in this study.

## Results

On April 20, 2021, the potential reach for our criteria (men with 1 or more of the following interests as defined by FB: “homosexuality, same-sex marriage, same-sex relationship, transgenderism, or LGBT community”) in the United States was approximately 16 million for all age groups and approximately 3.3 million for the age group 13 to 24 years.

### Age Group 13 to 24 Years

**Figure 5:**
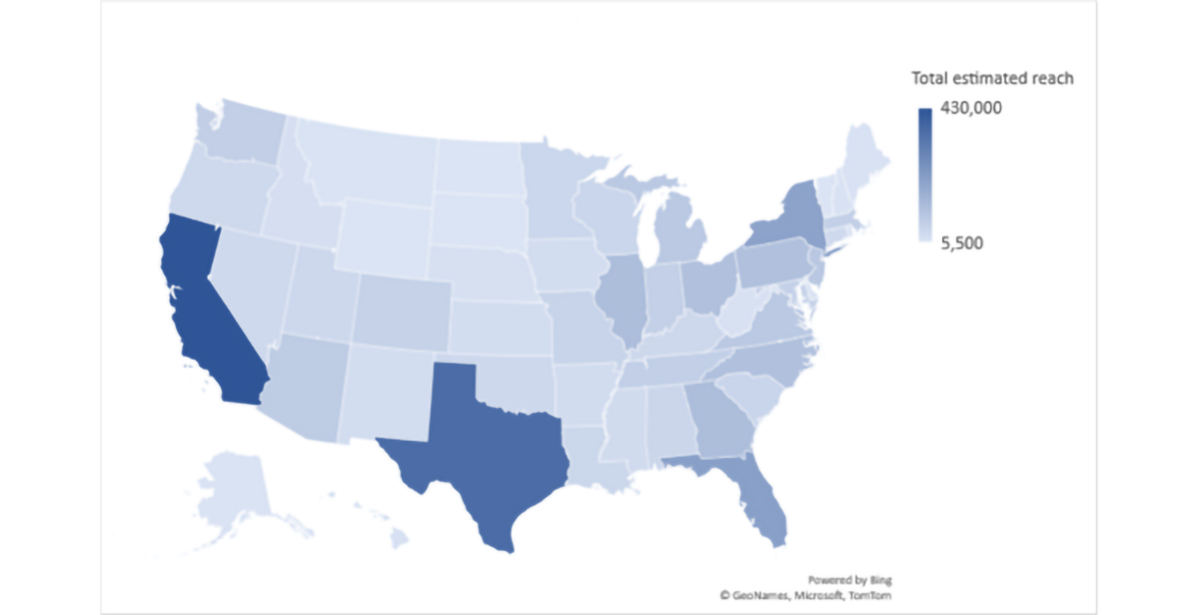
Estimated potential reach on Facebook per state on April 20, 2021, based on the target audience.

**Figure 6:**
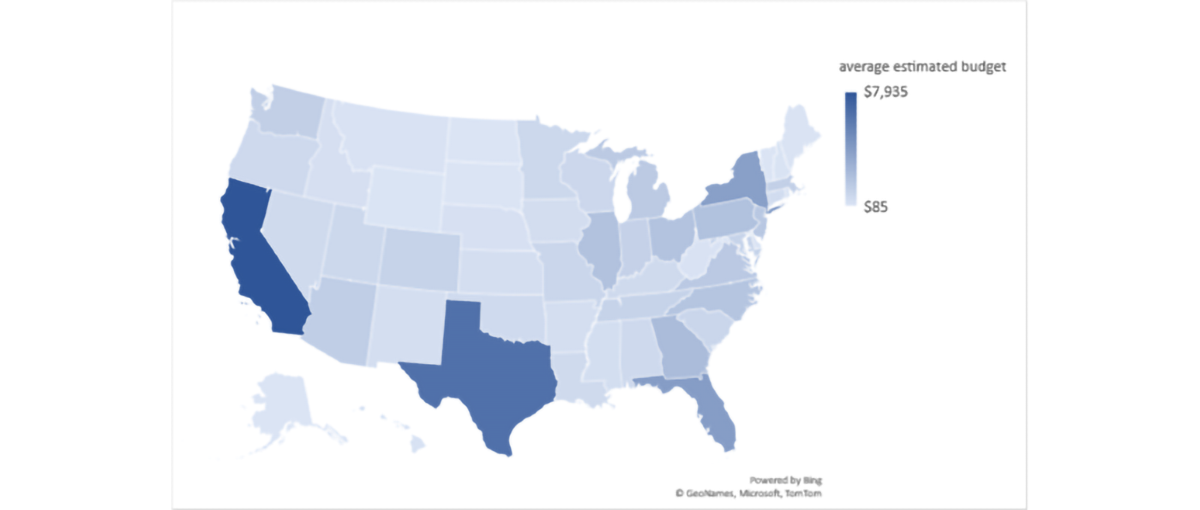
Average estimated Facebook Ads budget per state, manually adjusted to reach the potential reach based on target audience in each state on April 20, 2021.

California had the highest potential reach at 430,000, followed by Texas (360,000), Florida (210,000), and New York (200,000; Table 2 and Figure 5). The average estimated ad budget to reach the maximum potential audience on FB through a 10-day video ad campaign from April 20, 2021, to April 30, 2021, was the highest in California (US $7935, US $18.45 per 1000), followed by Texas (US $6425, US $17.85 per 1000), Florida (US $3990, US $19.00 per 1000), and New York (US $3835, US $19.18 per 1000; Figure 6).

**Figure 7:**
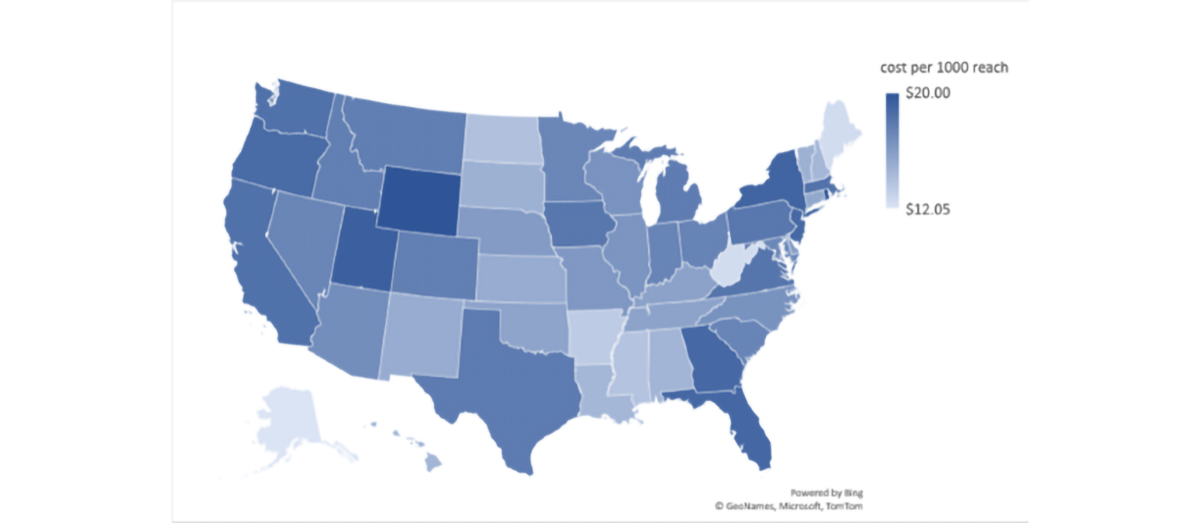
Average cost per 1000 reach on the Facebook Ads platform per state on April 20, 2021, based on target audience.

On the basis of the average estimated budget to reach the maximum audience for each state in the age group 13 to 24 years, the estimated average cost per 1000 individuals reached was the lowest in Alaska, Maine, and West Virginia (US $12-US $12.99), followed by Arkansas and Mississippi (US $13-US $13.99) and Alabama, Hawaii, Louisiana, New Hampshire, Ohio, South Dakota, and Vermont (US $14-US $14.99). The estimated average cost per 1000 individuals reached was the highest in Rhode Island and Wyoming at US $20, followed by Florida, New York, and Utah (US $19-US $19.99) and California, Georgia, Iowa, Massachusetts, New Jersey, Oregon, Pennsylvania, Virginia, and Washington (US $18-US $18.99; Figure 7).

**Figure 8:**
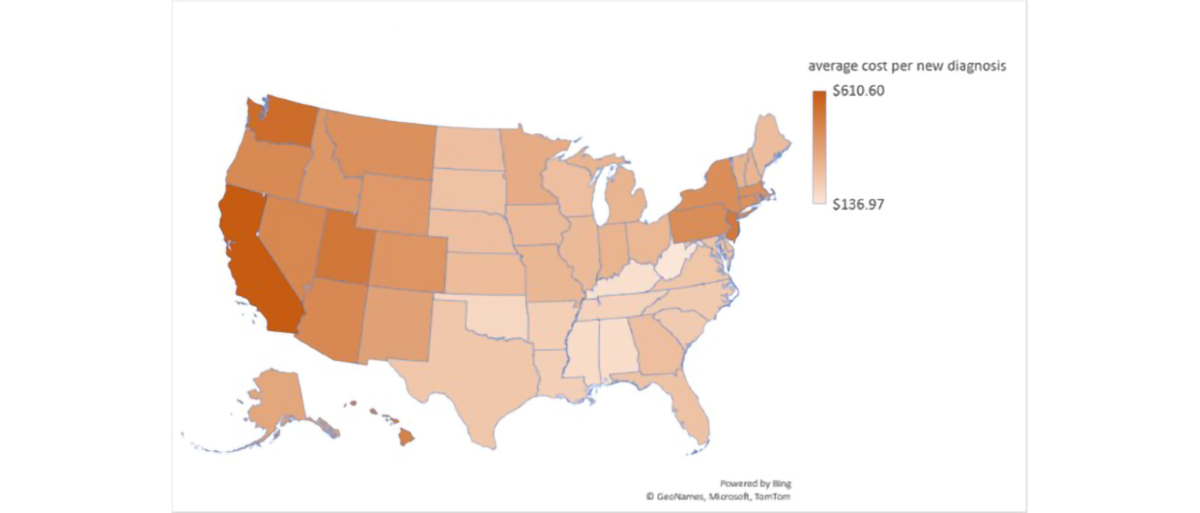
Average Facebook Ads cost per new HIV diagnosis per state as simulated on April 20, 2021, based on Facebook potential reach, Facebook estimated link clicks, average health care industry conversion rate, and the Centers for Disease Control and Prevention-reported average positivity rate per US regions.

The average FB ad cost for every new HIV diagnosis was the lowest in Alabama, Kentucky, Mississippi, Oklahoma, and West Virginia (US $136.97-US $199.99), followed by Arkansas, Delaware, Louisiana, North Carolina, South Carolina, Tennessee, Texas, and Virginia (US $200-US $249.99) and Florida, Georgia, Indiana, Iowa, Kansas, Maine, Maryland, Missouri, Nebraska, North Dakota, Ohio, South Dakota, and Wisconsin (US $250-US $299.99). The highest estimated average ad’s cost for every new HIV diagnosis was found in California at US $610.60, followed by Washington (US $547.99), Utah (US $515.02), and New Jersey (US $508.03; Figure 8).

Using a single ad with the same target criteria for the entire United States demonstrated an estimated average cost per 1000 of US $18.50 and an estimated average cost per new HIV diagnosis of US $257.51.

**Table 2:** Estimated total reach, daily reach, and daily link clicks on the Facebook Ads platform based on the selected target criteria for age group 13 to 24 years and the manually adjusted budget to include the total reach on both extreme ends of the provided estimated range of reach.

State	Total estimated reach	Lowest adjusted budget (US $)	Highest adjusted budget (US $)	Estimated lowest daily reach at the lowest budget	Estimated highest daily reach at the lowest budget	Estimated lowest daily reach at the highest budget	Estimated highest daily reach at the highest budget	Estimated lowest daily link clicks at the lowest budget	Estimated highest daily link clicks at the lowest budget	Estimated lowest daily link clicks at the highest budget	Estimated highest daily link clicks at the highest budget
Alabama	46,000	200	1150	1600	4700	4600	13,200	50	146	114	330
Alaska	8300	30	170	287	830	831	2400	5	19	16	46
Arizona	78,000	450	2180	2700	7800	7800	22,500	67	194	158	456
Arkansas	29,000	120	660	998	2900	2900	8300	21	61	52	152
California	430,000	3440	12,430	14,900	43,000	43,000	124,200	288	832	718	2100
Colorado	59,000	290	1790	2000	5900	5900	16,900	56	162	140	405
Connecticut	35,000	170	910	1200	3500	3500	10,000	26	77	65	188
Delaware	9400	60	240	345	997	940	2700	8	22	17	48
Florida	210,000	1520	6460	7300	21,000	21,000	60,600	187	539	435	1300
Georgia	120,000	810	3730	4100	12,000	12,000	34,600	98	284	244	704
Hawaii	14,000	60	350	476	1400	1400	3900	9	25	25	71
Idaho	19,000	100	570	688	2000	1900	5400	20	59	44	127
Illinois	120,000	560	3390	4100	12,000	1200	34,500	120	347	285	823
Indiana	64,000	320	1900	2200	6500	6400	18,400	64	185	149	431
Iowa	26,000	140	800	914	2600	2600	7400	30	88	66	190
Kansas	26,000	120	670	926	2700	2600	7400	25	72	57	165
Kentucky	41,000	190	1100	1400	4100	4100	11,800	50	145	115	331
Louisiana	45,000	200	1110	1600	4500	4500	12,900	38	110	86	249
Maine	12,000	40	260	452	1300	1200	3400	12	35	27	78
Maryland	61,000	340	1760	2100	6100	6100	17,600	47	136	120	347
Massachusetts	64,000	360	1990	2200	6400	6400	18,400	57	165	141	406
Michigan	86,000	420	2640	3000	8600	8600	24,700	86	250	207	599
Minnesota	44,000	190	1330	1600	4500	4400	12,600	36	104	97	280
Mississippi	31,000	130	730	1100	3100	3100	8800	30	88	71	205
Missouri	54,000	240	1520	1900	5400	5400	1550	49	142	124	357
Montana	9900	50	300	349	1000	1000	2900	10	28	22	64
Nebraska	18,000	80	500	616	1800	1800	5100	19	54	44	127
Nevada	37,000	200	1080	1300	3700	3700	10,600	32	92	77	222
New Hampshire	11,000	60	260	409	1200	1100	3000	12	37	25	73
New Jersey	88,000	670	2650	3000	8800	8800	25,300	69	198	166	481
New Mexico	26,000	120	670	884	2600	2600	7400	25	73	58	167
New York	200,000	1370	6300	6900	20,000	20,000	57,700	188	545	435	1300
North Carolina	110,000	600	3050	3800	11,000	11,000	31,700	98	283	224	648
North Dakota	6400	30	150	256	740	661	1900	6	19	13	38
Ohio	110,000	520	3310	3800	11,000	11,000	31,700	113	327	266	770
Oklahoma	40,000	190	1060	1400	4000	4000	11,400	41	117	95	274
Oregon	37,000	230	1160	1300	3700	3700	10,600	35	101	85	246
Pennsylvania	110,000	650	3330	3800	11,000	11,000	31,700	93	268	232	670
Rhode Island	11,000	60	380	375	1100	1100	3100	11	31	28	82
South Carolina	49,000	250	1450	1700	4900	4900	14,100	43	124	108	311
South Dakota	6700	30	170	318	918	684	2000	7	22	15	44
Tennessee	67,000	310	1790	2300	6800	6700	19,200	61	176	148	428
Texas	360,000	2240	10,610	12,400	36,000	36,000	104,000	316	913	751	2200
Utah	40,000	260	1290	1400	4000	4000	11,500	33	97	84	242
Vermont	5700	30	140	251	726	591	1700	6	20	13	38
Virginia	85,000	530	2570	2900	8500	8500	24,400	76	221	179	518
Washington	68,000	470	2040	2400	6800	6800	19,600	51	146	128	369
West Virginia	14,000	50	300	495	1400	1400	3900	15	44	37	106
Wisconsin	48,000	220	1370	1700	4800	4800	13,700	51	147	120	346
Wyoming	5500	30	190	195	564	567	1600	6	18	15	44
Sum all states	3,294,900	19,750	95,960	114,334	330,875	318,774	933,950	2896	8388	6941	20,196
All United States^a^	3,300,000	17,480	104,590	114,200	330,000	330,000	953,600	3700	10,700	9300	27,000

**^a^**One single advertisement for all US for same target audience.

### Exploring Different Ad Budgets

**Figure 9:**
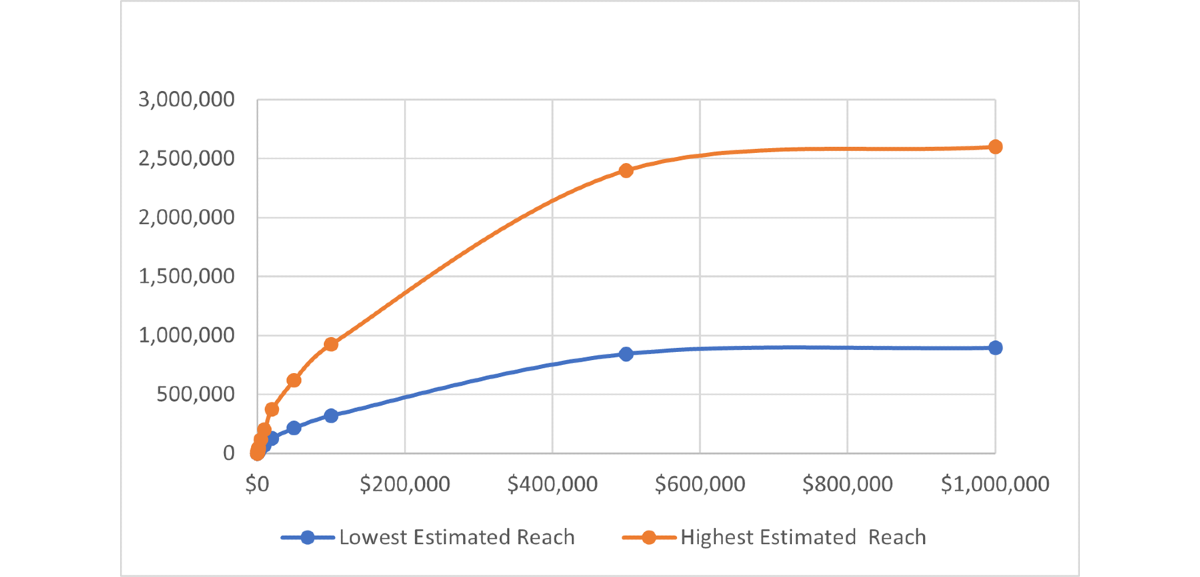
Cumulative distribution function curve of exponential distribution that “reach per budget” on the Facebook (FB) advertisement (ad) platform follows based on simulated different budgets for same target audience with same ad settings.

**Figure 10:**
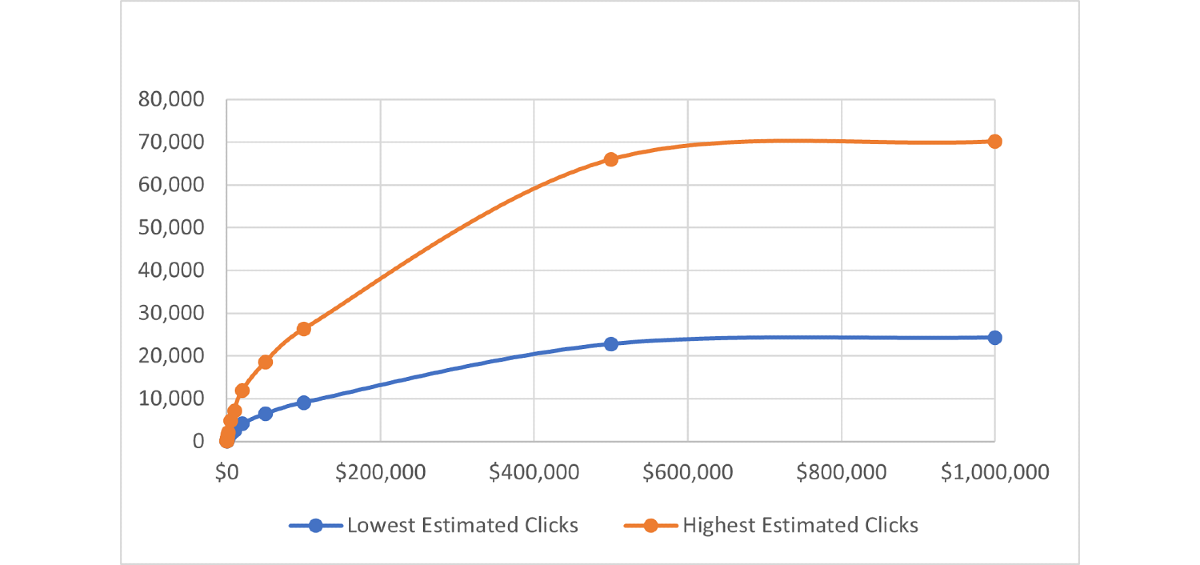
Cumulative distribution function curve of exponential distribution that “clicks per budget” on Facebook (FB) advertisement (ad) platform follows based on simulated different budgets for same target audience with same ad settings.

When we created a single ad using the criteria for the entire United States at different budgets ranging from US $10 to US $1,000,000, the daily reach per dollar and daily clicks per dollar followed the cumulative distribution curve of an exponential function (Figures 9 and 10).

Using an estimated reach of 16 million active US FB users on April 20, 2021, the ad cost per new HIV diagnosis for a 1-time national ad ranged from US $72.76 to US $452.25, with an average cost of US $166.79. In contrast, the cost range for every new HIV diagnosis with a campaign using multiple daily US $10 ads over 10 days was lower at US $13.09 to US $37.82 with an average cost of US $19.45. The estimated daily clicks per US $10 were also higher when using multiple daily US $10 ads and averaged 28 clicks per day compared with 3 daily clicks per US $10 when using a single US ad.

### Across Multiple Age Groups

Using multiple US $10 ads for our target audience in the United States across multiple age groups, the lowest average estimated cost per new HIV diagnosis was in the age groups 20 to 29 and 30 to 39 years (US $13.96 and US $14.72, respectively). Potential reach was the highest in the age group 20 to 29 years at 5.4 million, followed by the age group 30 to 39 years at 3.8 million and 3.2 million for the age group greater than 50 years. Potential reach was the lowest for the age group 13 to 19 years at 890,000. Estimated daily clicks using US $10 ten-day campaign ads were similar for all age groups (31-33), except for the age group 13 to 19 years where the estimated daily clicks were lower at 11. The estimated ad’s cost per HIV test averaged US $0.28 to US $0.29 for all age groups, except the age group 13 to 19 years, for which the estimated ad’s cost per HIV test averaged US $0.83. The estimated ad’s cost per HIV diagnosis averaged US $13.96 to US $34.97 for all age groups, except the age group 13 to 19 years that averaged US $55.10 (Table 3).

**Table 3:** Estimated advertisements (ads) cost per new HIV diagnosis for different age groups and geographic locations based on previously reported HIV positivity rates and estimated total reach on Facebook for the target audience of each age group and geographic location.

Targeting characteristics		New HIV positivity test percentage^a^	Potential reach on Facebook	Estimated positive tests	Estimated ads cost per new HIV diagnosis (US $)
**Age at test (years), n**					
	13-19	1.50	890,000	58	55.10
	20-29	2.10	5,400,000	1298	13.96
	30-39	1.90	3,800,000	712	14.72
	40-49	1.30	2,200,000	261	22.20
	50+	0.80	3,200,000	166	34.97
**Region, n**					
	Northeast	1.30	2,700,000	337	24.11
	Midwest	1.60	2,900,000	390	19.26
	South	2.30	6,200,000	1339	12.55
	West	1.20	3,800,000	508	25.25
	US dependent areas	1.10	150,000	7	24.67
	Total	1.70	16,000,000	2115	19.45

^a^Centers for Disease Control and Prevention funded HIV testing: United States, Puerto Rico, and the United States Virgin Islands, 2017 [28].

### FB Ad Cost Per US Regions

Using multiple US $10 ads for the target audience in different US regions, the lowest average estimated cost per new HIV diagnosis was in the South (US $12.55), followed by the Midwest (US $19.26). Potential reach was highest in the South at 6.2 million, followed by the West at 3.8 million, 2.9 million in the Midwest, 2.7 million in the Mideast, and 150,000 in the US dependent territories. The estimated daily clicks using US $10 ten-day campaign ads were similar for all regions at 29-34. The estimated ad cost per HIV test averaged US $0.27 to US $0.31 for all US regions and was the lowest in the US dependent territories at US $0.27. The estimated ads cost per HIV diagnosis averaged US $12.55 to US $24.67 for all US regions and was the lowest in the South at US $12.55 (Table 3).

## Discussion

### Evaluating Efficacy and Cost of FB Ads Reaching Population at Risk for HIV

Creating FB ads that target at-risk populations for HIV infection in US states, regions, and age groups while comparing different ad budgets revealed interesting and useful findings, including potential low ad cost per new HIV diagnosis and higher cost efficiency for low-budget ads. On April 20, 2021, the potential reach for our target criteria in the United States was approximately 16 million for all age groups and 3.3 million for the age group 13 to 24 years—the group that had the highest rate of undiagnosed HIV infection. The estimated ad cost per new HIV diagnosis averaged US $13.96 to US $55.10 for all age groups and was the highest in the age group 13 to 19 years at US $55.10. The estimated ad cost per new HIV diagnosis averaged US $12.55 to US $24.67 for all US regions and was the lowest in the South at US $12.55. Comparing different ad budgets, low-budget ads were more cost-effective than high-budget ads, as the daily reach per US dollar and the daily clicks per US dollar followed the cumulative distribution curve of an exponential function on the FB ad platform. We concluded that multiple small campaigns would generate similar results to larger campaigns at a lower cost.

Understanding the variables contributing to the cost and efficacy of social media ads for health promotion is important in the digital era [33]. Our study methodology proposes a framework (Figure 4) for estimating the efficacy and cost of FB ads per new diagnosis before launching the ads. At our study time, examining social media metrics among the US states revealed the highest estimated potential reach and the highest estimated ad budgets in California and Texas (Figures 5 and 6). However, the average cost per 1000 reach on the FB ad platform (Figure 7) was not the highest in California or Texas, which is likely owing to a proportionally lower ads cost in these 2 states at the time of our study. Moreover, the average FB ad cost per new HIV diagnosis (Figure 8) was the lowest in Texas and the highest in California. This difference was likely related to a difference in the ads cost between the 2 states and higher HIV test positivity rates in Texas than in California.

Although our framework focuses on FB ads, evaluating the platform’s potential as a health communication solution should be the first step when considering social media for health promotion. Selecting the best social media platform for health promotion campaigns can be accomplished by understanding the available criteria for targeting, comparing reach estimates of the target audience on the social media platform to real-world estimates of the population at risk, and examining the penetration rates of different social media platforms among the population of interest. For example, in our study, among all age groups, we found the highest FB ad cost per new HIV diagnosis in the age group 13 to 19 years. This may be explained by the lower penetration of FB among this age group than other platforms such as TikTok and Snapchat. In addition, these estimates are subject to user interaction with the ad’s design and content, which is beyond the scope of this study.

Since the emergence of the HIV epidemic, US health departments, community organizations, and health care providers have helped people with HIV become aware of their diagnosis and live longer. Timely, active, and complete surveillance is the mainstay of effective public health action, as undiagnosed, acute HIV infection carries an increased risk of complications and spread. As social media can help identify and stop microepidemics, the Joint United Nations Programme on HIV or AIDS highlighted the use of social media to facilitate community engagement as a critical component in HIV control efforts [34]. Our exploratory study provides a single snapshot in time and demonstrates the use of targeted FB ads to empower communities to reach at-risk populations for HIV efficiently. Using the ads platform, we could share educational messages regarding HIV risk factors while directing individuals to nearby HIV testing sites, thereby facilitating engagement in HIV care or HIV prevention (eg, PrEP) based on test results.

Owing to the global economic crisis in 2008, a major reduction in HIV prevention resources occurred at the state, local, and federal levels. To counteract the effect of decreased funding, the CDC published a guide on high-impact prevention to maximize the effect of limited resources [35]. Proven HIV prevention interventions include HIV testing and linkage to care, antiretroviral therapy, access to condoms and sterile syringes, prevention programs for people with HIV and their partners, prevention programs for people at high risk for HIV infection, substance use disorder treatment, and screening and treatment for other sexually transmitted infections in addition to PrEP [35]. Considering the high potential reach, granular targeting criteria selection, and relatively low cost compared with other conventional methods, FB ad campaigns can be an effective outreach tool for HIV prevention interventions.

Despite the decrease in the annual number and rate of HIV diagnoses in the United States from 2015 to 2019 [4], diagnoses remain unevenly distributed among the US regions. The South continues to have the highest rates of HIV infection at 15.2 per 100,000 people compared with the Northeast, West, and Midwest at 9.4, 9.2, and 7.2, respectively [3]. FB ads targeting at-risk populations for HIV in the South had the highest total potential reach and the lowest cost per new HIV diagnosis among all US regions. The high reach and low cost per new diagnosis make FB ads an attractive method for public health authorities to tackle the HIV epidemic in the South, where there is a disproportionate burden of disease. Similarly, considering the high potential reach for at-risk populations for HIV in all US regions at an average ad cost of US $12.55 to US $25.25 for 1 new HIV diagnosis, leveraging targeted FB ads can facilitate reaching the goal of ending the HIV epidemic in this decade [5].

As public health efforts to reach HIV-positive individuals are hampered by financial constraints, an understanding of cost becomes crucial to investing in appropriate resources. In 2013, the CDC designed the “Start Talking. Stop HIV.” page on FB (as part of a larger “ACT against AIDS” campaign) to reach and influence gay and bisexual men to spark conversations about HIV prevention and sexual health [36]. To date, this FB page has generated over 125,000 followers while only spending US $11,943 for ads between May 7, 2018, and February 7, 2022 [37]. In our study, the reach per dollar on FB ads followed a cumulative exponential distribution curve. We want to alert public health agencies that a single high-cost ad is more expensive and may have less impact than same-value multiple low-cost ads. In our study, using multiple lower cost FB ads, we estimated an average of US $19.45 for 1 new HIV diagnosis in the United States and as low as US $12.55 in the Southern states. In contrast, using 1 single national ad resulted in a cost of US $166.79 per new HIV diagnosis.

For future implementation of our simulated models, ethical considerations targeting at-risk populations for HIV who are frequently marginalized and disadvantaged should include community advisory groups empowered by representatives of the proposed recipients of the ads. This will promote engagement in the development of the study design and ad’s content to accomplish inclusion of the targeted participants for intervention while balancing the concern of representation that may have a further stigmatizing effect. In addition, future implementation designs should ensure the protection of participants’ privacy while targeting vulnerable populations, which may generate an identifiable digital trail.

Since our study was conducted, multiple social media platforms have changed their policies to prevent targeting users younger than 18 years with disinformation. In addition, FB no longer allows targeting sexual orientation interests, which may hinder public health outreach efforts on the platform for sexual health education. Considering the challenge of misinformation spreading via social media platforms and the resulting societal harm [14], it stands to argue that social media platforms owe it to the society to counteract disinformation. The authors of this paper argue that social media platforms should partner with public health organizations to provide a free outreach window for spreading health education information similar to their response during the COVID-19 pandemic.

### Limitations

Our study has several limitations that must be discussed. First, during the ad creation process for each state, the manually adjusted budget was rounded up to the closest US $10, which may falsely increase the estimated cost per click, cost per 1000 reach, and cost per new HIV diagnosis, especially in the states with the lowest potential reach. Second, we did not use the average health care industry CTR in our estimations, given that CTR is usually based on impressions, and the platform only provides estimated reach and estimated clicks during the process of ad creation. If estimated impressions were available during the creation of ads, we would have used the industry CTR to calculate estimated clicks from impressions and performed subsequent simulations. Third, potential reach, estimated daily reach, and estimated daily clicks were collected on the same day to avoid fluctuations that occurred based on different ads bidding and active FB users in the prior 30 days to data collection. All these estimates may change substantially when using a different time range given the fluctuations associated with the ad platform seasonality. Fourth, in our simulations, we used the positivity rates published in the CDC-funded HIV testing report from 2017, which has the most recently reported US data for test positivity rates for different age groups and US regions, and it may be different from the 2021 estimates. Fifth, we used the health care industry average conversion rate on FB as reported by WordStream based on a sample of 256 US clients in all industries between November 2016 and January 2017, which may also differ from the 2021 average health care conversion rate. Finally, our proposed feasibility model is subject to the limitations and algorithms of the social media platform because it is heavily based on estimates provided by the FB ad platform during the process of ad creation. FB ad estimates are based on multiple factors, including ad bids and active FB and Instagram users, may be intentionally misleading, and are not designed to match the census population.

### Conclusions

Targeted FB ads have the potential to reach populations at risk for HIV and to facilitate education on exposure risks, HIV testing, and PrEP. This is especially critical in the Southern United States given the increased rates of new HIV diagnoses, high potential reach of FB, and low cost per new HIV diagnosis. Although FB allows targeted and granular location-based and interest-based ads at varying budgets (critical for public health interventions), our study found that multiple small-budget ads were more cost-efficient than 1 large-budget ad campaign. From a public health perspective, this translates into local FB ads being more economical than a single large-budget national FB ad. Therefore, our future efforts will leverage these findings by focusing on populations at risk for HIV in the Dallas-Fort Worth metroplex through different FB ads targeting strategies to delineate the best combination of interest- and location-based targeting criteria to improve HIV care.

## Appendix

Multimedia Appendix 1 Data published and supplementary data available [37].

## Abbreviations

ad: advertisement
CDC: Centers for Disease Control and Prevention
CTR: click through rate
FB: Facebook
LGBT: lesbian, gay, bisexual, and transgender
PrEP: pre-exposure prophylaxis

## References

[1] Watson RJ, Collibee C, Maksut JL, Earnshaw VA, Rucinski K, Eaton L (2022). High levels of undiagnosed rectal STIs suggest that screening remains inadequate among Black gay, bisexual and other men who have sex with men. Sex Transm Infect.

[2] UNAIDS.

[3] HIV surveillance reports. Centers for Disease Control and Prevention, 2019.

[4] (2021). Estimated HIV incidence and prevalence in the United States 2010–2019. Centers for Disease Control and Prevention.

[5] Monitoring Selected National HIV Prevention and Care Objectives by Using HIV Surveillance Data United States and 6 Dependent Areas, 2020. Centers for Disease Control and Prevention.

[6] Shi L, Tang W, Hu H, Qiu T, Marley G, Liu X, Chen Y, Chen Y, Fu G (2021). The impact of COVID-19 pandemic on HIV care continuum in Jiangsu, China. BMC Infect Dis.

[7] Ridgway JP, Schmitt J, Friedman E, Taylor M, Devlin S, McNulty M, Pitrak D (2020). HIV care continuum and COVID-19 outcomes among people living with HIV during the covid-19 pandemic, Chicago, IL. AIDS Behav.

[8] Petersen C, Lehmann CU (2018). Social media in health care: time for transparent privacy policies and consent for data use and disclosure. Appl Clin Inform.

[9] Medford RJ, Saleh SN, Sumarsono A, Perl TM, Lehmann CU (2020). An "Infodemic": leveraging high-volume twitter data to understand early public sentiment for the coronavirus disease 2019 outbreak. Open Forum Infect Dis.

[10] Saleh SN, Lehmann CU, Medford RJ (2021). Early crowdfunding response to the COVID-19 pandemic: cross-sectional study. J Med Internet Res.

[11] Saleh SN, Ajufo E, Lehmann CU, Medford RJ (2020). A comparison of online medical crowdfunding in Canada, the UK, and the US. JAMA Netw Open.

[12] Saleh SN, Lehmann CU, McDonald SA, Basit MA, Medford RJ (2021). Understanding public perception of coronavirus disease 2019 (COVID-19) social distancing on Twitter. Infect Control Hosp Epidemiol.

[13] Wilson AE, Lehmann CU, Saleh SN, Hanna J, Medford RJ (2021). Social media: a new tool for outbreak surveillance. Antimicrob Steward Healthc Epidemiol.

[14] Lanier HD, Diaz MI, Saleh SN, Lehmann CU, Medford RJ (2022). Analyzing COVID-19 disinformation on Twitter using the hashtags #scamdemic and #plandemic: retrospective study. PLoS One.

[15] Diaz MI, Hanna JJ, Hughes AE, Lehmann CU, Medford RJ (2022). The politicization of ivermectin tweets during the COVID-19 pandemic. Open Forum Infect Dis.

[16] Number of monthly active Facebook users worldwide as of 3rd quarter 2022. Statista.

[17] Ads targeting. Facebook.

[18] Wozney L, Turner K, Rose-Davis B, McGrath PJ (2019). Facebook ads to the rescue? Recruiting a hard to reach population into an internet-based behavioral health intervention trial. Internet Interv.

[19] Kebede MM, Pischke CR (2019). Popular diabetes apps and the impact of diabetes app use on self-care behaviour: a survey among the digital community of persons with diabetes on social media. Front Endocrinol (Lausanne).

[20] Guillory J, Wiant KF, Farrelly M, Fiacco L, Alam I, Hoffman L, Crankshaw E, Delahanty J, Alexander TN (2018). Recruiting hard-to-reach populations for survey research: using Facebook and Instagram advertisements and in-person intercept in LGBT bars and nightclubs to recruit LGBT young adults. J Med Internet Res.

[21] Ramo DE, Rodriguez TM, Chavez K, Sommer MJ, Prochaska JJ (2014). Facebook recruitment of young adult smokers for a cessation trial: methods, metrics, and lessons learned. Internet Interv.

[22] Dehlin JM, Stillwagon R, Pickett J, Keene L, Schneider JA (2019). #PrEP4Love: an evaluation of a sex-positive HIV prevention campaign. JMIR Public Health Surveill.

[23] Burke RR, Weichelt BP, Namkoong K (2021). Facebook ads manager as a recruitment tool for a health and safety survey of farm mothers: pilot study. JMIR Form Res.

[24] Ford KL, Albritton T, Dunn TA, Crawford K, Neuwirth J, Bull S (2019). Youth study recruitment using paid advertising on Instagram, Snapchat, and Facebook: cross-sectional survey study. JMIR Public Health Surveill.

[25] Staffileno BA, Zschunke J, Weber M, Gross LE, Fogg L, Tangney CC (2017). The feasibility of using Facebook, craigslist, and other online strategies to recruit young African American women for a web-based healthy lifestyle behavior change intervention. J Cardiovasc Nurs.

[26] Gittelman S, Lange V, Gotway Crawford CA, Okoro CA, Lieb E, Dhingra SS, Trimarchi E (2015). A new source of data for public health surveillance: Facebook likes. J Med Internet Res.

[27] Chunara R, Bouton L, Ayers JW, Brownstein JS (2013). Assessing the online social environment for surveillance of obesity prevalence. PLoS One.

[28] Mejova Y, Weber I, Fernandez-Luque L (2018). Online health monitoring using Facebook advertisement audience estimates in the United States: evaluation study. JMIR Public Health Surveill.

[29] Meta for business. Facebook.

[30] Irvine M Facebook Ad Benchmarks for YOUR Industry [Data]. WordStream.

[31] (2017). CDC-Funded HIV Testing: United States, Puerto Rico, and the U.S. Virgin Islands, 2017. Centers for Disease Control and Prevention.

[32] Jero J OFBADSHIV. GitHub.

[33] Hanna J, Nijhawan A, Lehmann C, Medford R (2022). Simulating Facebook advertisements to establish cost per new HIV diagnosis using routine and targeted models in a local population. Healthcare (Basel).

[34] Understanding and acting on critical enablers and development synergies for strategic investments. United Nations Development Programme.

[35] High-Impact HIV Prevention CDC’s Approach to Reducing HIV Infections in the United States. Centers for Disease Control and Prevention.

[36] High-Impact HIV Prevention. HIV social media resources. Centers for Disease Control and Prevention.

[37] Start Talking. Stop HIV. Facebook.

