# Supplementary material for: Reaching Populations at Risk for HIV Through Targeted Facebook Advertisements: Cost-Consequence Analysis
Source: JMIR Form Res. 2023 Jan 20;7:e38630. doi: 10.2196/38630 (PMC9898830; doi:10.2196/38630)
Supplement: Multimedia Appendix 1 [file formative_v7i1e38630_app1.docx]

## Multimedia Appendix 1:

Data published and supplementary data available at <https://github.com/johnjero/OFBADSHIV/>
